# Adapt or Die: Targeting Unique Transmission-Stage Biology for Malaria Elimination

**DOI:** 10.3389/fcimb.2022.901971

**Published:** 2022-06-09

**Authors:** Mariëtte E. van der Watt, Janette Reader, Lyn-Marié Birkholtz

**Affiliations:** ^1^ Institute for Sustainable Malaria Control, School of Health Systems and Public Health, University of Pretoria, Pretoria, South Africa; ^2^ Department of Biochemistry, Genetics and Microbiology, University of Pretoria, Pretoria, South Africa

**Keywords:** antimalarials, gamete, gametocyte, malaria, *Plasmodium*, sexual commitment, transmission blocking

## Abstract

*Plasmodium* parasites have a complex life cycle that includes development in the human host as well as the *Anopheles* vector. Successful transmission of the parasite between its host and vector therefore requires the parasite to balance its investments in asexual replication and sexual reproduction, varying the frequency of sexual commitment to persist within the human host and generate future opportunities for transmission. The transmission window is extended further by the ability of stage V gametocytes to circulate in peripheral blood for weeks, whereas immature stage I to IV gametocytes sequester in the bone marrow and spleen until final maturation. Due to the low gametocyte numbers in blood circulation and with the ease of targeting such life cycle bottlenecks, transmission represents an efficient target for therapeutic intervention. The biological process of *Plasmodium* transmission is a multistage, multifaceted process and the past decade has seen a much deeper understanding of the molecular mechanisms and regulators involved. Clearly, specific and divergent processes are used during transmission compared to asexual proliferation, which both poses challenges but also opportunities for discovery of transmission-blocking antimalarials. This review therefore presents an update of our molecular understanding of gametocyte and gamete biology as well as the status of transmission-blocking activities of current antimalarials and lead development compounds. By defining the biological components associated with transmission, considerations for the development of new transmission-blocking drugs to target such untapped but unique biology is suggested as an important, main driver for transmission-blocking drug discovery.

## Introduction

Amongst infectious diseases, malaria has caused one of the most longstanding global health burdens ever. The impact of malaria on public health systems and socio-economic growth remains hard felt in several developing countries, with the World Health Organization (WHO) Africa region still carrying 94% of the global malaria burden. Concerted global efforts aimed towards malaria elimination did result in a 28% reduction in global malaria morbidity and 43% reduction in mortality, with countries such as Algeria, Argentina, China, and El Salvador now newly certified as malaria-free. Unfortunately, aside from these successes, global progress has stalled since 2015. In 2020 alone, there were approximately 241 million cases in 85 malaria-endemic countries with an estimated 627 000 deaths (WHO, 2021). Alarmingly, increases in both incident rates (9%) and the number of fatalities (12%) were observed year-on-year due to compounding factors, not least of which were disruptions experienced in clinical services caused by the ongoing COVID-19 pandemic (WHO, 2021). Although most of the gains against this disease are the result of highly effective vector control strategies, the efficacy of these is also under threat and effective elimination of the disease must rely on multipronged approaches. These include using tools to target the parasite pool as causative agent for disease, with innovations including vaccines such as RTS,S recently approved by the WHO for use in children under five (Laurens, 2020), and using antimalarial agents for additional applications aside from their continued chemotherapeutical use. This importantly also includes transmission-blocking capabilities, although the majority of clinically used antimalarials do not have this ability mostly due to differences associated with the variant biology of the different life cycle forms of malaria parasites (Burrows et al., 2017; Birkholtz et al., 2022). This therefore motivates strategies towards the discovery of transmission-blocking antimalarial agents, focused on targeting unique transmission-associated biology; both as the focus of this review.

## *Plasmodium* spp. and Transmission to Mosquitoes

Infectious diseases caused by parasites represent some of the most complex and complicated biological systems, the least of which is malaria. This disease requires intricate interplay of three entities – the human host (with all its genotypic and social complexities), ~40 dominant malaria transmission species of *Anopheles* mosquitoes and parasitic protists of the genus *Plasmodium*. Not only can six species of *Plasmodium* cause various severity of disease in humans [including *P. falciparum*, *P. vivax, P. malariae, P. ovale* (with subspecies *P. ovale curtisi* and *P. ovale wallikeri*), and zoonotic *P. knowlesi* and *P. cynomolgi*, (Ngotho et al., 2019)], but these parasites have some of the most complex life cycles yet characterized.

**Figure 1:**
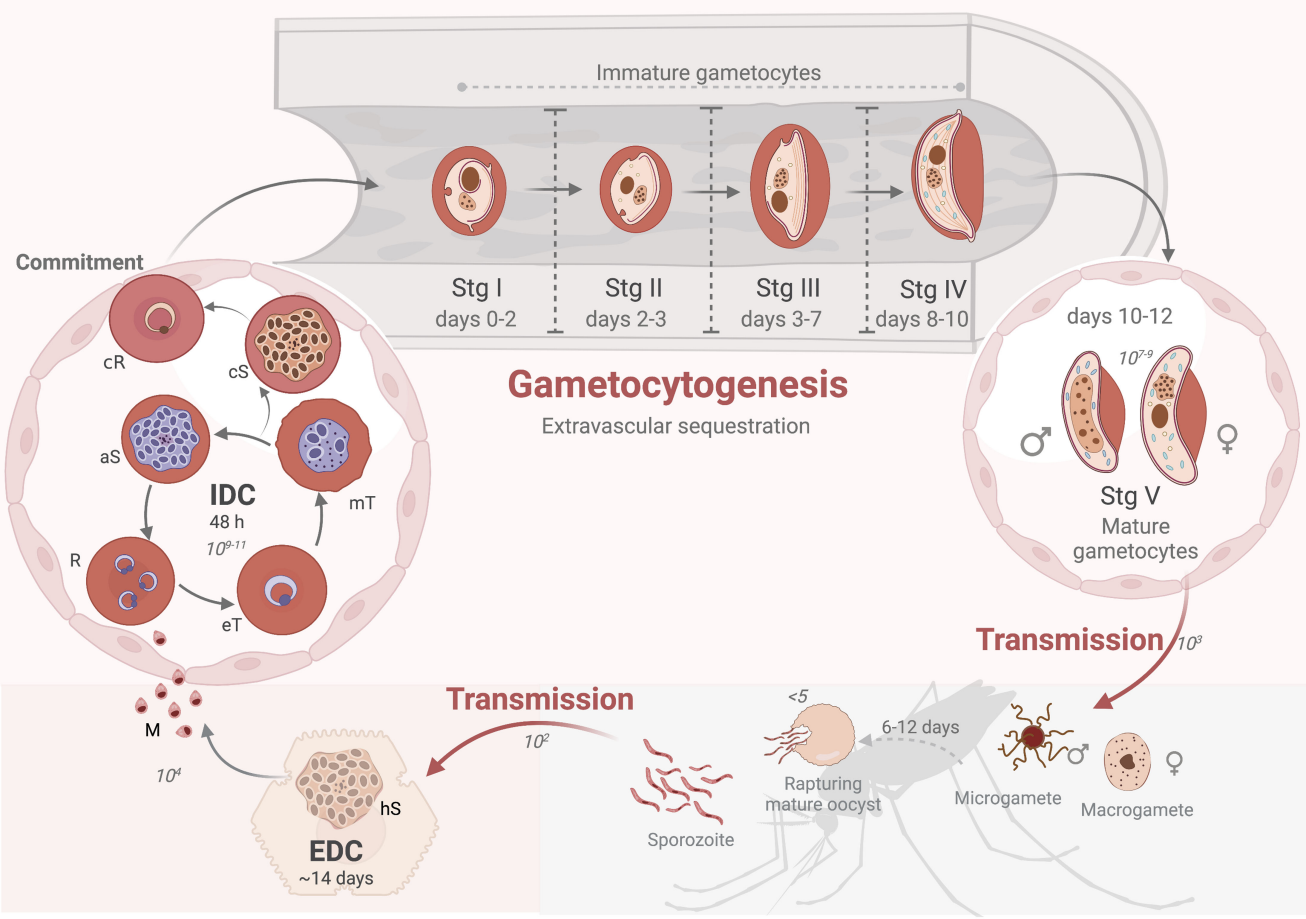
The *Plasmodium falciparum* life cycle. The life cycle has three broad phases: sexual reproduction in the mosquito vector (grey shaded area), asexual reproduction in the liver (exo-erythrocytic developmental cycle, EDC, in dark pink) and asexual reproduction (intra-erythrocytic developmental cycle, IDC) and gametocyte differentiation in erythrocytes (light pink). Transmission to humans is initiated by ~10^2^ sporozoites, which migrate to the liver through peripheral circulation to liver cells and replicate to form hepatic schizonts (hS). Hepatic asexually committed merozoites (M), released into the bloodstream, infect erythrocytes to initiate the IDC where parasites develop through ring (R), early trophozoite (eT), mature trophozoite (mT) and asexual schizont (aS) in 48 h, resulting in 10^11^ parasites. Gametocyte-committed schizonts (cS) results in either male or female committed rings (cR) that mature in extravascular spaces (e.g., bone marrow). *P. falciparum* gametocytes have five distinct morphological stages during development, stage I-IV as immature gametocytes taking ~10 days to form and ~10^7-9^ mature male and female stage V gametocytes returning to the circulatory system (~days 10-12), to be transmitted (~10^3^ gametocytes) to the mosquito. This is followed by gamete activation, fertilization, and production of an oocyst for sporogony. Created and modified with BioRender.com.

An infection in humans is initiated when as few as 100 sporozoites of the 10^3^ available in the salivary gland of a feeding female *Anopheles* mosquito are injected into the skin of a human as it takes a blood meal (Graumans et al., 2020). These sporozoites are the result of sporogony in a mature oocyst that formed in an infective mosquito after 6-12 days (**Figure 1**). Upon inoculation, sporozoites glide through the dermis into peripheral blood circulation within 30 min and migrate to the liver sinusoids to infect hepatocytes within 2 min, followed by the initiation of exo-erythrocytic schizogony (Prudencio et al., 2006). This characterizes the first obligate intracellular auxotroph stage as a true hallmark of parasitism. Succeeding ~14 rounds of replication in the liver during the asexual, exo-erythrocytic developmental cycle (EDC, **Figure 1**), mass cytokinesis occurs followed by the release of ~10^4^ of hepatic merozoites from a single sporozoite into the bloodstream. Blood stage infection is established when these merozoites invade erythrocytes, thus initiating the asexual 48 h intra-erythrocytic developmental cycle (IDC, **Figure 1**). A single, haploid merozoite evolves into a ring-stage parasite within 6 h post-invasion (hpi) (Butler et al., 2014; Van Biljon et al., 2018), followed by development into metabolically active trophozoites (Teng et al., 2009). Schizogony commences when the single trophozoite nucleus begins to divide into two daughter nuclear bodies around 33-36 hpi and results in a polyploid, multi-nucleated syncytium (Gerald et al., 2011; Van Biljon et al., 2018; Simon et al., 2021). Erythrocyte rupture occurs at 42-48 hpi, releasing 8-24 merozoites per schizont (Bannister and Mitchell, 2003; Gerald et al., 2011) and each released merozoite proceeds to infect a new erythrocyte, allowing for persistent cycles of infection. This coordinated, rapid amplification results in a massive population expansion for the parasite, reaching up to 10^11^ parasites per infected, untreated, immune-naïve individual, and is enabled by the ability of the parasite to evade the immune system, efficiently use its intracellular microenvironment for cellular growth (‘tropho’ for nourishment) and asexual replication (schizogony). This highly coordinated replication cycle is associated with malaria pathology in humans.

Possibly the most extraordinary and important biological feat associated with the parasite’s life cycle is its ability to differentiate to ensure transmission, and therefore continued spread of the disease and guaranteed survival of the organism (**Figure 1**). In a terminal differentiative process, only a small sub-population of asexual parasites (<10%) commit to sexual differentiation during an IDC, thereby ensuring the remaining proliferating parasites are available to seed subsequent differentiation and cause continuous transmission. This initiates gametocytogenesis to ensure transmission, a process requiring only 10^3^ mature male and female gametocytes in an average 1-2 µL *Anopheles* blood meal. It is during this process that these gametocytes are activated, and male gametocytes respond to the changed environment to undergo an extraordinary 3 mitotic divisions, forming 8 axonemes in <20 min (in a process called exflagellation) as one of the best examples of the ability of eukaryotic cells to respond rapidly to signaling events (**Figure 1**). Although most *Plasmodium* spp. form gametocytes within ~24-48 h, in the most important human malaria parasite, *P. falciparum*, gametocytes uniquely develop through five morphologically, biochemically and physiologically distinct stages in 10-12 days (**Figure 1**) (Sinden et al., 1977; Dixon and Tilley, 2021). This prolonged development is unique to the *Laverania* [falciparum and reichenowi (Ngotho et al., 2019)]. Additionally, gametocytogenesis in *P. falciparum* is associated with tissue sequestration of immature forms around erythroblastic islands in e.g. the bone marrow parenchyma (Venugopal et al., 2020), which is mechanistically different to asexual parasite infected erythrocyte sequestration to host cells in both *P. falciparum* and other rodent spp (Tiburcio et al., 2013). Lastly, prolonged survival of mature forms [mean lifespan ~5.5 days, *in vivo* circulation of up to 55 days (Bousema et al., 2010)] also conceptually contributes to the sustained transmission success that is achieved by *P. falciparum*.

*P. falciparum* stage I gametocytes resemble the rounded, asexual trophozoite but can be distinguished from these since hemozoin crystals do not form punctate clusters in stage I gametocytes (Brancucci et al., 2018) and there are no knobs on the host cell (**Figure 1**). Stage II gametocytes mark the start of drastic morphological changes, and these adopt a lemon shape with mononuclear content. As stage II to stage III transition occurs, the gametocyte (including its nucleus and mitochondria) elongates to a length to width of ratio ~2:1, facilitated by extensive ultrastructural changes, with one side flattening while the opposite side curves forming an elongated D-shape (top hat shape) (Parkyn Schneider et al., 2017; Dixon and Tilley, 2021). The nuclear elongation (maximal in stage III, retracts in stage V) is clear from 3D analysis performed through serial block-face scanning electron microscopy (EM) (Parkyn Schneider et al., 2017) and reiterated in a study of gametocyte nuclear pore proteins, visualized by fluorescence microscopy (Boltryk et al., 2021). Sexual dimorphism now also becomes more pronounced with small differences microscopically observable between the sexes, including the larger nucleus of the male and the cytoplasm of the female now containing more mitochondria (detectable through fluorescent probes) and extensive endoplasmic reticulum (ER), Golgi vesicles and dense spherules (the latter observed by electron microscopy) compared to the male. Stage IV gametocytes are maximally elongated with a crescent banana shape (length to width ratio of 4:1) with aciculate ends; this differentiates them from mature stage V male and female gametocytes (macro- and microgametocytes). These retain the characteristic falciform crescent shape from which the species falciparum derives its name (‘falx’ meaning sickle or curved shape and ‘parere’ to ‘bring forth’). These mature stages have characteristic rounded ends and a length to width of ratio of 3:1; the host erythrocyte is reduced to a thin background layer referred to as a Laveran’s bib. Female gametocytes are distinguished from males as they are slightly more curved and have concentrated nuclear material, well developed ER, mitochondria and apicoplast in preparation for development as zygote. Membrane-bound osmiophilic bodies are visible in both sexes as distinct vesicle-like structures and allow release of the gametocytes from the erythrocytes during gametogenesis.

## Targeting Transmission for Malaria Elimination

Interventions that will inhibit the formation of gametocytes, and thereby transmission, are required to contribute to malaria elimination strategies (Leroy et al., 2014; Birkholtz et al., 2016; Sinden, 2017; Delves et al., 2018a; Birkholtz et al., 2022). There are multiple reasons why human to mosquito transmission of *Plasmodium* is an attractive target for intervention. Gametocytes represent a targetable population bottleneck (Sinden, 2017); they are present in the pharmacologically accessible blood compartment; mature, stage V gametocytes can persist for days and mature sexual parasites are extracellular for ~24 h in the mosquito, creating a significant window during which to target the parasite for therapeutic/immune destruction. Transmission-blocking antimalarials would drastically reduce the parasite reservoir (even in high-transmission settings), could target the significant proportion of asymptomatic parasite carriers and, possibly the most enticing, could protect the lifespan of antimalarials that kill asexual blood stages (ABS) if used in combination, by preventing the spread of resistant parasites against the ABS active antimalarial (Delves et al., 2018a; Birkholtz et al., 2022).

Clear differentiation between *P. falciparum* gametocytes and ABS explains why most ABS antimalarials are inactive against mature gametocyte stages and therefore not useful to block transmission. Currently, whilst primaquine, methylene blue and atovaquone can target malaria transmission, each have concerns ranging from toxicity in certain populations (Howes et al., 2012; Goncalves et al., 2016) to a very narrow target parasite population (e.g., atovaquone targeting ookinetes). The search for new compounds targeting the transmissible stages have been skewed towards compounds targeting biology important to proliferation of the ABS and large screening campaigns have mostly prioritized hits based on ABS activity as primary filter – with transmission-blocking activity seen as advantageous additional activity for dual-active antimalarials. However, if the same molecular activity is targeted in both ABS and gametocytes/gametes, and resistance develops against such antimalarial hit, spread of resistance is a real threat (Witmer et al., 2021). Alternative strategies towards identifying antimalarial hits with transmission-blocking activity include *de novo* screening against either mature gametocytes or gametes (Miguel-Blanco et al., 2017; Delves et al., 2018b; Reader et al., 2021). This has revealed novel chemotypes associated with targetable biology in these stages. In principle, our broader understanding of the unique biology of gametocytes and gametes (Delves, 2012; Meibalan and Marti, 2017; Josling et al., 2018; Ngotho et al., 2019; Schneider and Reece, 2021; Usui and Williamson, 2021) therefore present a major, unexplored avenue to discover new antimalarials, and with the toolkit associated with transmission-blocking screens now well established (Delves et al., 2018a; Birkholtz et al., 2022), these should be exploited with renewed effort (Sinden et al., 2012; Leroy et al., 2014; Nilsson et al., 2015; Delves et al., 2018a). Open areas worth investigating include the commitment phase, early differentiation processes during immature gametocytogenesis, maturation of stage V gametocytes and gametogenesis itself, although the latter will rely on irreversibly compromising (sterilizing) mature stage V gametocytes, as these will be the target of intervention in humans.

## Commitment to Sexual Differentiation

Commitment to sexual differentiation marks the key decision point at which the parasite responds to signals to ‘adapt or die’ i.e., to transmit or not. This irreversible and binomial decision is variable and can take place within the same IDC cycle, whereby merozoites entering erythrocytes either directly initiate gametocytogenesis at low frequency (same cycle conversion), or complete another round of asexual replication after which daughter merozoites released from the committed schizont invade erythrocytes and only then initiate gametocytogenesis (next cycle conversion) (Bancells et al., 2019). This depends on the temporal expression of factors responsible for sexual differentiation. In either situation, the merozoite progeny of a single schizont will become either all male or all female gametocytes, resulting in the biased production of committed schizonts and the 4:1 (female:male) sex ratio of *P. falciparum* (Silvestrini et al., 2000; Smith et al., 2000).

Commitment is a clear example of eukaryotic processes employed by *Plasmodium* in response to external factors, the majority of which are related to host or environmental stressors that the parasite can sense and that forces it into sexual commitment in a classic mechanism of species survival (Paul et al., 2002; Talman et al., 2004; Sowunmi et al., 2008). These signals include high parasitemia or increased pathology experienced in the host [anemia or hemolysis, high lymphocyte or reticulocyte densities (Gautret et al., 1996; Trager et al., 1999), and immune pressure (Ono et al., 1986)] or external factors including co-infections (Menezes et al., 2018). But nutrient sensing is one of the main external factors inducing commitment. LysoPC restriction (a phosphatidyl choline, PC, abundant in serum) induces gametocyte production *in vitro* (Brancucci et al., 2017) due to the complete reliance of ABS on PC for conversion into lipid membranes through the Kennedy pathway. When LysoPC is depleted, ABS parasites can only survive for a single asexual cycle by using phosphoethanolamine (PE) methyltransferase (*Pf*PMT) to produce PC *via* the trimethylation of PE (Witola et al., 2008; Brancucci et al., 2018).

Molecular role players downstream of LysoPC sensing that regulate gametocytogenesis have been somewhat elucidated and include *P. falciparum* gametocyte development 1 (*Pf*GDV-1) as the key role player in gametocytogenesis, specific to *P. falciparum* and divergent from rodent *Plasmodium* spp. (Brancucci et al., 2017). *Pf*GDV-1 regulates expression of the transcription factor *Pf*AP2-G from the apicomplexan-specific Apetala2 transcription factor family (Eksi, 2012; Filarsky et al., 2018), by evicting heterochromatin protein 1 (*Pf*HP1) from the histone post-translational modification (PTM) H3K9me3 site upstream of *ap2-g* (Filarsky et al., 2018), a mark that is maintained by histone deacetylase 2 (*Pf*Hda2). *Pf*GDV-1 is itself regulated by an antisense RNA mechanism (Broadbent et al., 2015), and AP2-G can regulate its own transcription through feedback inhibition (Kafsack et al., 2014; Sinha et al., 2014). A key outstanding question is how these metabolites link to the maintenance of heterochromatin that controls the frequency of *ap2-g* activation. One connection is the requirement of *S*-adenosylmethionine (SAM) for both histone methylation (H3K9me3) and PE methylation by *Pf*PMT. With *Pf*PMT likely consuming 3x the amount of SAM under PC restricting conditions, H3K9me3 may not be maintained (Neveu et al., 2020a), thereby lifting the heterochromatic restriction on the *ap2-g* locus.

### Can Sexual Commitment be Targeted?

Commitment to gametocytogenesis can seemingly be increased *in vivo* due to drug treatment including the presence of steroid hormones (Lingnau et al., 1993), fansidar (Puta and Manyando, 1997), chloroquine, sulphadoxine-pyrimethamine (Talman et al., 2004) and other antimalarials (Peatey et al., 2009; Portugaliza et al., 2020). This has often raised a concern as to potential increased transmission of the parasite under sub-optimal treatment conditions. Indeed, quantitative data on sexual commitment rates after drug (including antimalarials) exposure indicated elevated sexual commitment as a general stress response at a narrow window around the IC_50_ and in the case of mefloquine and pyrimethamine, resulted in a net increase in gametocyte production (Thommen et al., 2022). Whether this translates to therapeutic induction of gametocytes is unclear, but it would be a concern if antimalarials are not used at effective doses, as can often be the case in some endemic regions.

The question then remains if there are any druggable processes during commitment that would prevent the initiation of the evolutionary escape process? With the involvement of *Pf*PMT, the Kennedy pathway and epigenetic modulators, one could foresee potential for small molecule interventions. However, beyond the above evidence of drugs inducing gametocytogenesis when used on ABS parasites, no reports of drugs preventing gametocyte commitment by targeting the molecular role players are available, possibly due to the difficulty associated with detecting committed ring and schizont stages that precludes large scale investigations. The development of new *P. falciparum* reporter lines to detect commitment (Thommen et al., 2022) may open up investigations of new antimalarial leads blocking this process. Genetic manipulation have indeed resulted in several gametocyte deficient cell lines [e.g. *gdv1* (Tiburcio et al., 2021), *ap2-g* (Kafsack et al., 2014) and *pmt* (Bobenchik et al., 2011)], validating the importance of these regulators to gametocytogenesis and opening up potential avenues for intervention. Enzymes involved in *Plasmodium* spp. Kennedy pathway are of interest. *Pf*PMT can be inhibited on a protein level by MMV019918 (Vallone et al., 2018), hexadecyltrimethylammonium, dodecyltrimethylammonium and amodiaquine (Bobenchik et al., 2010; Garg et al., 2015) although whole cell inhibition data is lacking. Choline kinase (CK) is essential to *P. berghei* blood stages (Aoyama et al., 2004; Witola et al., 2008; Dechamps et al., 2010) and structural mimics of choline cause selective inhibition of CK (Witola and Ben Mamoun, 2007; Alberge et al., 2009).

Based on the strong involvement of epigenetic regulation of *Pf*AP-2G activation and the key H3K9me3/ac balance, it would be interesting to investigate if inhibitors of e.g. histone lysine methyltransferases (HKMTs) such as BIX01294 (that prevents ABS proliferation and gametocyte viability [Malmquist et al., 2015; Coetzee et al., 2020)], histone demethylases such as JIB-04 or ML324 [active against mature gametocytes (Matthews et al., 2020; Reader et al., 2021)], or hydroxamate-based HDACi (with potent antiplasmodial activity and limited cytotoxicity (Coetzee et al., 2020; Vanheer et al., 2020), also prevent gametocyte conversion and commitment.

Whether targeting any of these processes will in any form translate to compounds with therapeutic advantage is questionable. Indeed, such a compound will not clear ABS parasites, and will not prevent gametocyte maturation and transmission, unless commitment can be entirely halted before same cycle conversion occurs. Sole inhibition of commitment is therefore not a justifiable profile, and commitment-blocking activity will likely only be considered as an additional advantage of compounds with ABS activity, although the contribution of such added activity to overall efficacy (either as ABS cure or transmission-blocking active) is difficult to quantify.

## The Early Phase of Gametocyte Differentiation and Development

Once committed to gametocytogenesis, the early phase of gametocyte differentiation is marked by massive ultrastructural and functional adaptations in the associated immature (stage I-IV) gametocytes. Importantly, to avoid splenic clearance and elude the host immune system, immature gametocytes sequester in the extravascular space (erythroblastic islands) of the bone marrow parenchyma and in the spleen (Neveu et al., 2020b). The bone marrow provides a nutrient-rich, anaerobic environment with the relative distribution of fatty acids, including LysoPC, in the bone marrow, that differs greatly from that in serum (Brancucci et al., 2017).

**Figure 2:**
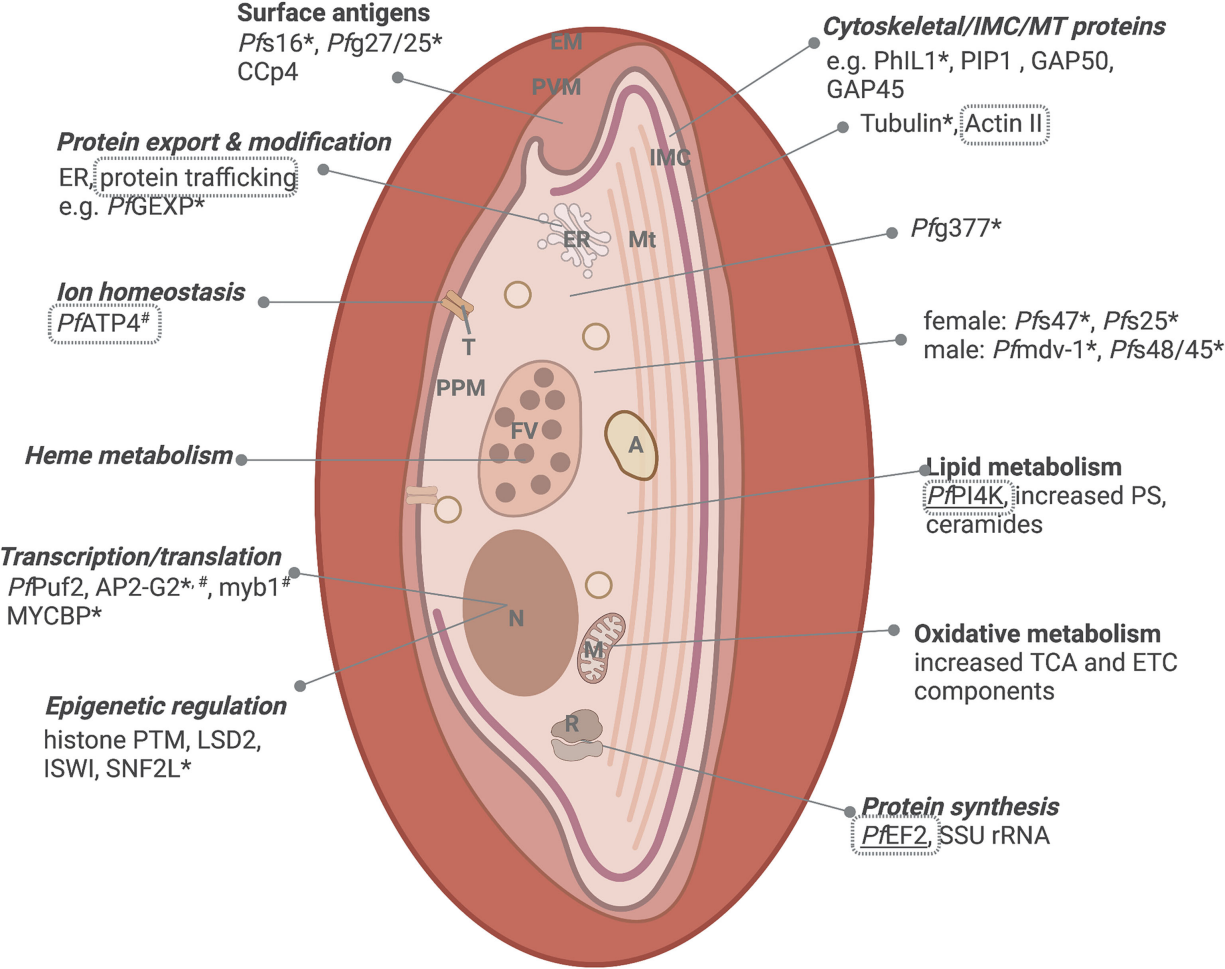
Important biological processes of immature gametocytes. Immature gametocytes require extensive transcriptional reprogramming, metabolic adaptations, and ultrastructural changes. Processes with evidence of targetability is indicated either through genetic validation of essentiality (*essential to gametocytes, ^#^essential to ABS, www.phenoPlasm.org; those genes refractory to manipulation indicating essentiality are underlined) or by chemical inhibition (grey text borders) and those shared with ABS are indicated in italics. EM, erythrocyte membrane; PVM, parasitophorous vacuolar membrane; IMC, inner membrane complex; ER, Endoplasmic reticulum; Mt, microtubular network; A, acidocalcisome; T, transporter; PPM, parasite plasma membrane; FV, food vacuole; N, nucleus; M, mitochondrion and R, ribosome. Protein names are explained in text. Created and modified with BioRender.com.

Morphologically, early stage I-III *P. falciparum* gametocytes are characterized by the increased presence of food vacuoles, extended polyribosomes, and the deposition of a network of sub-pellicular inner membranes (the inner membrane complex, IMC), to form the characteristic D-shape of stage III, subtended by a dense deposition of microtubules (MT), actin and formin-1 (**Figure 2**) (Dixon and Tilley, 2021). The IMC and MT network associates with glideosome components (GAP50, GAP45 and MTIP) and membrane trafficking proteins, PhIL1 and PIP (Parkyn Schneider et al., 2017). Immature *P. falciparum* gametocytes further actively remodel the host erythrocyte, thereby exposing antigens for cell-cell interactions (Messina et al., 2018), distinct from those used by ABS. Active protein export in *P. falciparum* immature gametocytes is exemplified by the upregulation of gametocyte exported proteins (GEXP) (Silvestrini et al., 2010), a third of which belong to the *Plasmodium* helical interspersed subtelomeric (PHIST) protein family (Sargeant et al., 2006) with known roles in host cell remodeling and protein trafficking (Warncke et al., 2016). Immature gametocytes also export rhoptry proteins to increase permeability and activate (reversibly) new permeation pathways (NPP) that mediate nutrient uptake in immature gametocytes (Bouyer et al., 2020). Taken together, these changes result in increased stiffness in the immature gametocyte to allow mechanical retention in the bone marrow and avoid splenic clearance.

On a molecular level, immature gametocytes are substantially distinguished from ABS by divergent sex- and stage-specific transcriptomes (Lopez-Barragan et al., 2011; Lasonder et al., 2016; Van Biljon et al., 2019). *Pf*GEXP5 is the earliest detected gametocyte marker expressed in an AP2-G independent manner (Tiburcio et al., 2015), but AP2-G dependent gametocyte-specific markers include *Pf*s16, *Pf*g27/25, *Pf*g14.744, *Pf*g14.745, *Pf*g14.748 and ETRAMP10.3/PEG4 (Kafsack et al., 2014), in addition to 308 other markers expressed in committed schizonts and early gametocytes (Pelle et al., 2015). The molecular role players associated with transcriptional reprogramming during immature gametocyte development have been somewhat elucidated, and include transcription factors like AP2-G2 (essential to maturation in these immature (Singh et al., 2021b), whilst increased expression implicates factors such as *Pf*Myb1 and MYCBP (a c-Myc binding protein) in sex-specific gene regulation. Post-commitment epigenetic regulators include unique histone methylation repressive marks associated with repression of ABS related genes (Coetzee et al., 2017; Connacher et al., 2021; Von Gruning et al., 2022), the upregulation of putative chromatin remodelers like ISWI and SNF2L (Poran et al., 2017) and expansion of HP1 occupancy (Fraschka et al., 2018)and use of repressive histone PTMs (e.g. H3K36me2/3) for a resultant heterochromatic nature (Connacher et al., 2022). The conversion to commitment is further evident in changes in mRNA dynamics with subsets of gametocyte-specific transcripts stabilized in these early stages, and ABS related mRNAs undergoing active decay (Painter et al., 2017).

The transcriptional reprogramming manifests on a metabolic level, firstly to adapt the gametocyte to aerobic energy production and secondly, to enable lipid biosynthesis towards eventual fertilization. This switch is most likely due to the gametocytes’ move to the hypoxic hematopoietic bone marrow where host-derived glutamine is the main carbon source (Srivastava et al., 2017). Whilst ABS parasites rely mostly on glycolysis for energy production, during early gametocytogenesis, metabolism moves to become more fermentative and occurs partly through the tricarboxylic acid (TCA) cycle and aerobic energy production *via* the electron transport chain (ETC) (Macrae et al., 2013). This is exemplified by the upregulation of 15/16 transcripts involved in TCA metabolism (Lasonder et al., 2016; Van Biljon et al., 2019), the presence of a large, branched mitochondrion and a 7-fold increase in the activity of cytochrome b (Crofts, 2004). The gametocyte lipidome also differs significantly from other life cycle stages with a clear enrichment (>8-fold) in phosphatidylserine (PS), ceramides and dihydroceramides in gametocytes (Lamour et al., 2014; Gulati et al., 2015).

Male-specific proteins (those required for later genome replication in preparation for exflagellation) appear earlier at stage I/II development (Van Biljon et al., 2019). Sex-specific proteins include *Pf*g377, involved in osmiophilic body production produced from stage III and important to females (Alano et al., 1995; Severini et al., 1999; de Koning-Ward et al., 2008), *Pf*s47 – a 6-cysteine domain protein expressed exclusively in females from stage II (Van Schaijk et al., 2006) and *Pf*s25 and CCp4 as female-specific surface protein (Schneider et al., 2015; Meerstein-Kessel et al., 2018). Although *Pf*MDV-1/PEG3 (male development protein 1 and protein of early gametocyte 3, respectively) is expressed in both sexes and its expression is regulated by *Pf*AP2-G2 (Xu et al., 2020), disruption of *pfmdv-1* results in a dramatic reduction in functionally mature male gametocytes and partially perturbed mosquito infectivity (Furuya et al., 2005). Pfs48/45 is expressed from stage II (Lobo et al., 1999) and its ablation reduces male fertility (Van Schaijk et al., 2006) whereas *Pf*Puf2 is a regulatory protein whose deletion results in aberrant male gametocyte differentiation (Miao et al., 2010).

### Targeting Immature Gametocytes

Targeting the process of early gametocyte sequestration and differentiation could have a dramatic impact on transmission and provides motivation for further investigation into the mechanisms parasites use to establish themselves in the bone marrow microenvironment. The majority of antimalarials with ABS activity will retain their activity on immature gametocytes if a similar biology/protein is targeted e.g., hemoglobin metabolism, which is only active until stage III development (Plouffe et al., 2016). This could therefore prevent any development beyond the immature stages. However, in some cases (e.g., the 4-aminoquinolines targeting heme metabolism, which is inactive by stage IV), there is a loss in activity and the effective concentration targeting immature gametocytes is higher than that required to kill the ABS; in some cases this is even more pronounced in stage IV gametocytes (**Table 1**) (Plouffe et al., 2016). In this scenario, if any immature gametocytes are formed, and cannot be effectively targeted, they will continue to seed mature gametocytes. Since only 20 parasites/µL can to result in an infective mosquito (Usui and Williamson, 2021), this poses a high risk to transmission-blocking activity. Additionally, if the same biology is targeted between ABS and immature gametocytes, and resistance develops in the ABS stages to a particular antimalarial, this would be transferred to the immature stage, rendering the antimalarial completely ineffective as transmission-blocking active and resulting in spread of resistant parasites. Lastly, several ABS targeting antimalarials are completely ineffective against immature stages (e.g., pyrimethymine and atovaquone), and data on the activity of some frontrunner and development compounds like Ganaplacide [KAF156, an imidazolopiperazine targeting protein secretion (Kuhen et al., 2014)], P218 (a pyrimidine targeting dihydrofolate reductase [(Posayapisit et al., 2021)] and MMV183 [a panthothenate targeting acetyl coA synthetase (Schalkwijk et al., 2019)], are lacking.

**Table 1:** Summary of historical antimalarials, current front-runner and development compounds and novel hits with sub-micromolar *in vitro* activity against *P. falciparum* immature (stage II/III) gametocytes.

Compound	IC_50_ (nM)		Target / MoA	Ref
	Stg II/III	Stg IV		
Artemether	5	19	Hemoglobin metabolism and hemozoin formation	(Plouffe et al., 2016)
Artemisinin	20	37		
Artemisone	2	4		
Artesunate	4	49		(Coertzen et al., 2018)
DHA	3	21		(Klonis et al., 2011; Coertzen et al., 2018)
OZ277	4	8		(Giannangelo et al., 2020)
Artefenomel (OZ439)	5	2		(Giannangelo et al., 2020)
Amodiaquine	6	2456		(Plouffe et al., 2016)
AQ-13	33	6471		
Chloroquine	98	6250		
Hydroxychloroquine	131	6250		
Napthoquine	14	4167		
Piperaquine	14	4167		
Pyronaridine	10	2579		
Lumefantrine	13	599		
Mefloquine (+RS)	39	579		
Halofantrine	1	1509		
Quinidine	208	12500	Unknown	
Quinine	496	12500	Unknown	
Cipargamin (KAE609)	100^&^	–	*Pf*ATP4 / Na^2+^ homeostasis	(Rottmann et al., 2010; Plouffe et al., 2016)
KAF246	2	2		
M5717 (DDD498)	5	2	*Pf*EF2	(Baragana et al., 2015; Plouffe et al., 2016)
MMV390048	214	–	*Pf*PI4K	(Paquet et al., 2017)
KDU691	565	2327		(Mcnamara et al., 2013)
Methylene blue	15	12	Glutathione reductase	(Buchholz et al., 2008; Plouffe et al., 2016)
Pentamidine	591	813	Hemozoin formation	(Plouffe et al., 2016)
KAI407	636	329	Unknown	(Plouffe et al., 2016)
NSC158011	90*	–	PfPMT	(Bobenchik et al., 2013)
GNF179	64	9	Protein trafficking & secretory pathway	(Plouffe et al., 2016)
BIX01294	14	–	HKMT	(Coetzee et al., 2020)
Cycloheximide	640	477	Unknown	(Plouffe et al., 2016)

*Inhibition of gametocyte viability 5 μM (%).

^&^Inhibition of gametocyte viability 50 nM (%).

Several antimalarial frontrunners retain activity against immature gametocytes with inhibition of Na^2+^ homeostasis by targeting the cation transporting ATPase *Pf*ATP4 (e.g., Cipargamin, KAF246), protein synthesis through inhibition of elongation factor *Pf*EF2 (e.g. M5717) and the phosphatidylinositol (PI)-4 kinase (PfPI4K) inhibitor MMV390048 (Paquet et al., 2017), resulting in some of the most potent compounds in development currently (**Table 1**). Their almost equipotent activity between ABS and immature gametocytes is imminently useful to block gametocytogenesis. This is also thought to be mediated by the NPP, still active in immature gametocytes (Bouyer et al., 2020).

As immature gametocytes are associated with transcriptional reprogramming, metabolic adaptations, and ultrastructural changes, any of these processes become appealing from an inhibitory perspective (**Figure 2**). However, aside from the compounds with additional ABS activity, for which targets have been elucidated, very few target-specific indicators have been described in these immature stages. But extrapolations from genetic validation of the essential nature of some of the processes involved in these early phases of gametocytogenesis could provide an avenue worth exploring to validate druggability of these processes. For instance, the genetic validation of the importance of PhIL1, PIP1 (and 3) and α-tubulin II to early gametocyte structural changes (Parkyn Schneider et al., 2017; Dixon and Tilley, 2021), mark these as potential drug targets, although no evidence of successful inhibition of homologues to these structural proteins is available. By contrast, the actin filaments that polarize to the opposite ends of the cell and co-localize with the actin nucleation factor, formin-1, are sensitive to cytochalasin-D, affecting bone marrow and spleen sequestration in *P. berghei* gametocytes, suggesting actin is required for tissue homing and the gametocytes’ subsequent return to circulation (De Niz et al., 2018).

## Maturation of Gametocytes to Transmissible Forms

The ultimate step in gametocyte development and differentiation requires the parasite to mature to be transmissible. Stage V gametocytes therefore change in shape to become less rigid to allow the parasite to return to the blood circulatory system and secondly, they adapt metabolically to survive in the human host for prolonged periods. The deformability switch leading to the release of stage V gametocytes from bone marrow or splenic retention is mediated by loosening of the IMC and disruption/partial disruption of associated proteins and with the loss of the MT network and STEVOR proteins (Dearnley et al., 2012; Tiburcio et al., 2013; Nilsson et al., 2015; Dixon and Tilley, 2021).

**Figure 3:**
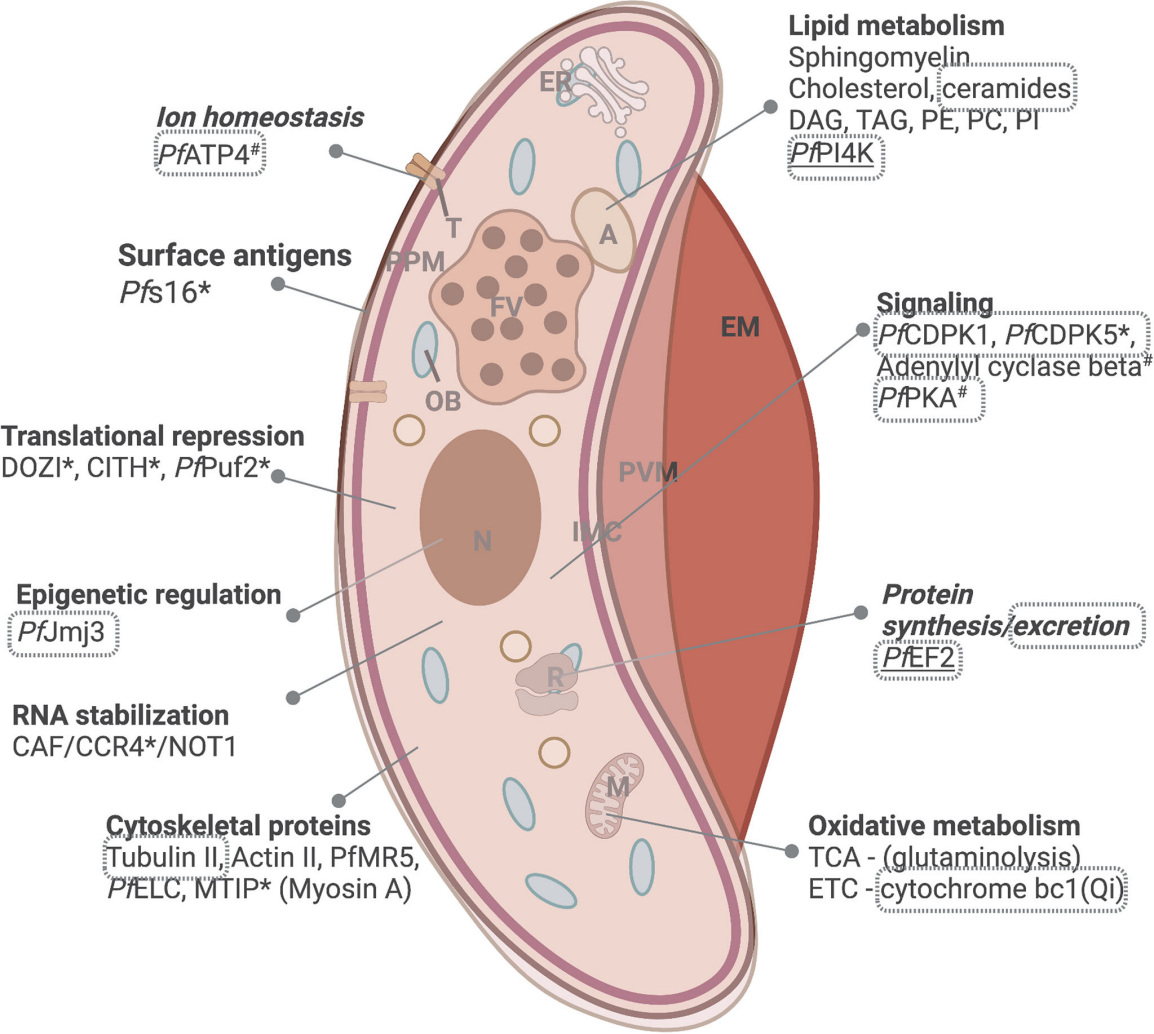
Distinct biology associated with mature gametocytes. Processes of importance include lipid metabolism, protein synthesis, signaling and oxidative metabolism, including mitochondrial respiration. Proteins that are essential to parasite survival (www.phenoPlasm.org) are indicated by a * (for gametocytes) or ^#^ (for ABS stages) and those indicated as druggable by chemical inhibition are indicated in grey bordered text. Genes refractory to manipulation indicating essentiality are underlined. EM, erythrocyte membrane; PVM, parasitophorous vacuole membrane; IMC, inner membrane complex; ER, Endoplasmic reticulum; A, acidocalcisome; OB, osmiophillic body; T, transporter; PPM, parasite plasma membrane; FV, food vacuole; N, nucleus; M, mitochondrion and R, ribosome. Protein names are explained in text. Created and modified with BioRender.com.

Metabolically, mature gametocytes do not metabolize hemoglobin and slow their flux through glycolysis. These parasites remain metabolically active but now, like typical eukaryotic cells, utilize glutaminolysis for oxidative metabolism through the TCA cycle for energy production (MacRae et al., 2013; Lamour et al., 2014) (**Figure 3**). This is underscored by distinct changes in the mature gametocyte transcriptome and metabolome. These shifts highlight the requirement of mature gametocytes to prepare for the challenging environment in the mosquito, where the energy demands are high, and efficiency is vital for transmission. Transcriptional reprogramming therefore results in ~20-25% of the *Plasmodium* genome expressed specifically in the sexual stages (Lasonder et al., 2016; van Biljon et al., 2019), creating an epigenetically-mediated transcriptionally poised state (Coetzee et al., 2017; von Gruning et al., 2022) including translational repression of female-associated transcripts (Lasonder et al., 2016). Regulators of translation like DOZI and CITH mediate the storage of mRNA transcripts in the form of ribonucleoprotein complexes within female gametocytes for rapid translation during female gamete activation. mRNA targets are bound by *Pf*Puf2, the disruption of which results in translation and the increased production of the associated protein (Miao et al., 2013). Those transcripts not stabilized are regulated by the CAF1/CCR4/NOT complex causing mRNA decay and thereby translational repression (**Figure 3**) (Hart et al., 2019; Hart et al., 2021).

Genes transcribed later in gametocytogenesis relate to the processes specific to maturation and include the switch from sequestration to peripheral circulation (deformability), as well as processes involved in readying the parasite for transmission to the mosquito (protein synthesis for locomotion, host cell entry and actin depolymerization) (van Biljon et al., 2019). The marked expansion of mitochondrial numbers and the exclusive presence of cristae observed in mature gametocytes (Evers et al., 2021) correlate with a 40-fold increase in ETC components (Evers et al., 2021) and upregulation of TCA associated genes (**Figure 3**) (Lasonder et al., 2016; van Biljon et al., 2019). Furthermore, vast changes in the lipid requirements in mature gametocytes ensure fluidity (Dearnley et al., 2012; Tibúrcio et al., 2012) and allow preparation for fertilization requiring membrane biogenesis, protein trafficking and cell signaling. This is evident in upregulation of actin depolymerization factors 1 and 2, leading to increased cellular deformability, enrichment of cholesterol esters and dihydrosphingomyelin in female gametocytes; the latter crucial for both sexes’ viability (Ridgway et al., 2022) and an up to 60-fold enrichment in diacylglycerol and triacylglycerol, associated with the presence of fatty acid-rich and osmiophilic bodies (including PE & PC) at the gametocyte periphery (Tran et al., 2016). These serve as energy storage to fuel the increases in protein and phospholipid biosynthesis during gametogenesis and subsequent fertilization (Besteiro et al., 2010). PI is essential for vesicle trafficking and as secondary messengers in gametocyte activation by mobilizing intracellular Ca^2+^ stores (Brochet et al., 2014). Other intracellular signaling role players include CDPK1, CDPK5, adenylyl cyclase beta and cAMP dependent protein kinase A (PKA-c and PKA-r) (van Biljon et al., 2019). Sex-specific transcripts upregulated in mature stages include cytoskeletal proteins α-tubulin II and actin II (Deligianni et al., 2011; van Biljon et al., 2019) and *Pf*MR5 (Eksi et al., 2006), both involved in the motility and viability of exflagellating gametes.

### Targeting Mature Gametocytes

Transmission-blocking requires compounds to either kill or substantially compromise (sterilize) mature gametocytes, such that drugs delivered to affected humans will prevent transmission (Birkholtz et al., 2022). However, most ABS actives – which typically target proliferative processes – are inactive on mature gametocytes, simply because the underlying biology is so markedly different or alternatively, uptake into mature gametocytes may be compromised due to inactive NPPs (Bouyer et al., 2020). Indeed, as indicated above, mature gametocytes are not quiescent but rather metabolically more like normal eukaryotic cells than the ABS, which are more alike cancerous cells with high metabolic flux through glycolysis and induction of the Warburg effect, resulting in fermentative states (Salcedo-Sora et al., 2014).

Protein synthesis, protein secretion, ion homeostasis and lipid metabolism remain powerful processes to target with M5717, GNF179, Cipargamin (KAE609) & KAF246, and MMV390048, respectively, displaying transmission-blocking activities at concentrations amenable to therapeutic indices (**Table 2**). However, if resistance develops against these compounds, it would likely be transmitted as the compounds would lose efficacy against mature gametocytes as well. This could be resolved by combination therapies where a compound targeting specific biology associated with gametocytes are combined with compounds targeting proliferative processes in ABS. Conceptually, this would protect the ABS component from resistance spread. However, with this strategy, it is essential to target unique biology in gametocytes. Such potential targets include the deformability of mature gametocytes, where the two light chains of the myosin motor *Pf*MyoA, *Pf*ELC and MTIP were shown to be essential and could be starting points for the development of novel antimalarials targeting the glideosome (Moussaoui et al., 2020) (**Figure 3**). Additional targets around deformability include cAMP signaling and STEVOR dephosphorylation with inhibition of phosphodiesterase by sildenafil citrate or PKA inhibition by KT5720 and H89 changing the flexibility of gametocytes (Ramdani et al., 2015). Ceramides and complex sphingolipids regulate membrane fluidity and increase in abundance during maturation. Two compounds targeting dhCer (ceramide precursor) synthase and displaying gametocytocidal activity were recently identified (Gulati et al., 2015), suggesting that lipid synthesis might be a viable target in mature gametocytes.

**Table 2:** Summary of key antimalarials, front-runner and development compounds as well as investigative hits with *in vitro* activity (< 5 µM) against *P. falciparum* mature gametocytes.

Compound	IC_50_ (nM) Stg V	Target	Ref
Mefloquine (+RS)	158	80S ribosome	(Plouffe et al., 2016)
ELQ-300	72	Cytochrome bc1(Qi)	(Nilsen et al., 2013)
Cipargamin (KAE609)	100*	*Pf*ATP4 / Na^2+^ homeostasis	(Van Pelt-Koops et al., 2012)
KAF246	2		(Rottmann et al., 2010; Plouffe et al., 2016)
M5717 (DDD498)	9	*Pf*EF2 / protein synthesis	(Rottmann et al., 2010; Plouffe et al., 2016)
MMV390048	140	*Pf*PI4K	(Paquet et al., 2017)
AZD-0156	236		(Reader et al., 2021)
KDU691	150		(Mcnamara et al., 2013)
ML324	77	*Pf*jmj3	(Reader et al., 2021)
JIB-04	3630		(Matthews et al., 2020)
Methylene blue	258	Glutathione reductase	(Buchholz et al., 2008; Plouffe et al., 2016)
GNF179	3	Protein secretory pathway	(Plouffe et al., 2016)
Birinapant	135	Unknown (caspase 3 inhibitor)	(Reader et al., 2021)
MMV1581558	130	Unknown (Adenosine A3 receptor)	
MMV1580843	108	Unknown	
SQ109	104	Unknown (MmpL3 in Mtb)	
Epoxomicin	6.6	β2 and 5 proteasome subunits	(D'Alessandro et al., 2016)

*% inhibition of gametocyte viability 500 nM.

Gametocyte-specific biology that is currently under investigation include a renewed focus on mitochondrial respiration, although the differentiation associated with TCA and the ETC in gametocytes have to be taken into account (Van Biljon et al., 2019; Evers et al., 2021), which explains the lack of activity of dihydroorotate inhibitors (e.g. DSM265). Targeting of cytochrome bc1 seems highly dependent on structural motifs of antimalarial compounds either biding the oxidative (Qo) site and a reductive (Qi) site (Fisher et al., 2020). Atovaquone binds the Qo site but this does not change gametocyte viability and atovaquone activity only manifests in ookinete formation (Jeevaratnam et al., 1992). However, targeting of the Qi site by ELQ-300 shows potent activity against mature gametocytes (**Table 2**). This potency emphasizes the importance of the TCA cycle in mature gametocytes for primary energy metabolism (Macrae et al., 2013; Lamour et al., 2014) and marking this as a prime targetable process, currently being investigated by the Medicine for Malaria Venture (Stickles et al., 2015a; Stickles et al., 2015b). Several other hit compounds are being used to explore such transmission-specific biology including histone demethylase inhibitors ML324 and JIB-04 (**Table 2**) (Matthews et al., 2020; Reader et al., 2021), and compounds with polypharmacology in protists including the protonophore SQ109 (Reader et al., 2021).

## Gametogenesis to Form Oocysts in the Mosquito Vector

In an extraordinary feat of biology, gametogenesis in *Plasmodium* is a result of finetuned responses to environmental signals associated with host to vector environmental changes, including a 5°C temperature drop, presence of xanthurenic acid and pH increase (7.2 to 8) (Arai et al., 2001). This results in rapid activation of DNA synthesis/replication, protein synthesis, axoneme assembly and egress within the male gametocyte, as well as protein synthesis and egress in the female gametocyte (Delves, 2012). Fertilization requires active motility, cell-to-cell recognition and membrane fusion, whereas ookinete development is dependent on vegetative growth, protein synthesis and motility (Delves, 2012).

**Figure 4:**
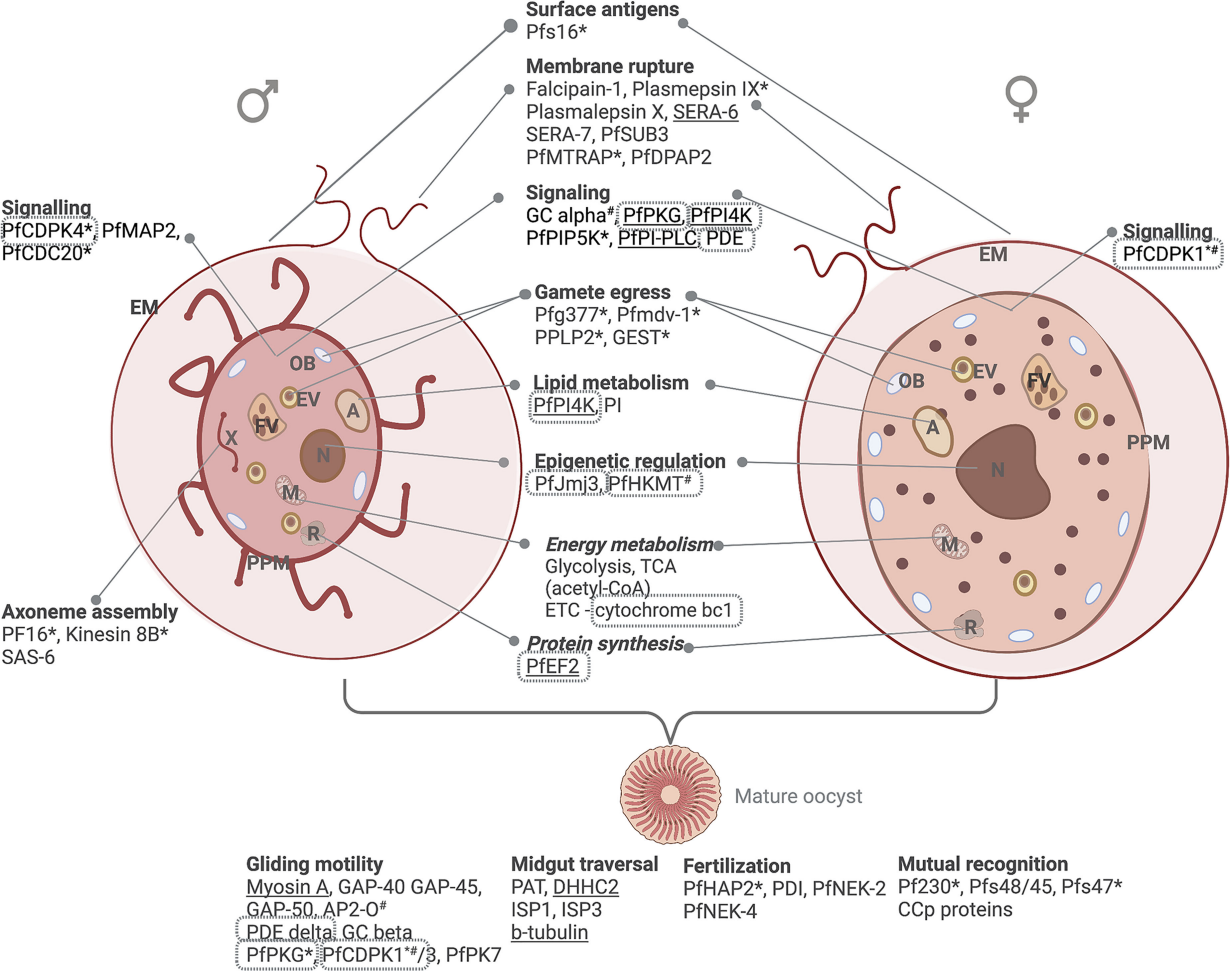
Male and female gametes and their differentiative biological processes. Gametogenesis involves intracellular signaling, DNA synthesis, lipid metabolism and energy metabolism reminiscent of the asexual stages (glycolysis and TCA cycle). Protein essentiality (www.phenoPlasm.org) is indicated by a * (gametocytes) or ^#^ (asexual stages) or underlined where the gene is refractory to deletion. Proteins that are validated as potential target through chemical inhibition are indicated in grey borders. EM, erythrocyte membrane; ER, Endoplasmic reticulum; A, acidocalcisome; EV, egress vesicle; OB, osmiophillic body; PPM, parasite plasma membrane; FV, food vacuole; N, nucleus; M, mitochondrion and R, ribosome. Protein names are explained in text.

The subsequent activation of guanylyl cyclase (GCα) and cyclic guanosine 3’, 5’-monophosphate (cGMP) signaling is crucial to activation of protein kinase G (PKG) as key role player in gametogenesis and egress (Kuehn and Pradel, 2010; Balestra et al., 2021), the signal transduction of which occurs, in part, through the multipass membrane protein ICM1 (Balestra et al., 2021) (**Figure 4**). The PKG mediated cascade (including involvement of *Pf*PI4K and phosphatidylinositol 4-phosphate 5-kinase, PIP5K) results ultimately in opening of IP_3_-gated calcium channels on the ER and a resultant increase in intracellular Ca^2+^ (Bennink et al., 2016). Activation of several sex-specific CDPKs ensues (Brochet et al., 2021), including CDPK1-mediated release of DOZI, CITH and *Pf*Puf2 for translation in female gametes and CDPK4 to activate cyclins and cyclin-dependent kinases for cell division in male gametes (Kumar et al., 2021) (**Figure 4**). The latter is mediated by successive S and M cell cycle phases (Raabe et al., 2009; Meibalan and Marti, 2017). The high energy demands of replicating, motile male gametes are satisfied through exclusive reliance on glycolysis (Talman et al., 2014; Srivastava et al., 2016).

Egress is mediated by the osmiophilic body proteins *Pf*g377, *Pf*MDV-1 and gamete egress and sporozoite survival (GEST) protein (De Koning-Ward et al., 2008; Bargieri et al., 2016), as well as the egress vesicle proteins, perforin-like protein (PPLP2) that mediates the formation of pores in the erythrocyte membrane (Sologub et al., 2011) (**Figure 4**). Cysteine and serine proteases (falcipain-1, plasmepsin IX, plasmalepsin X, SERA-6, SERA-7, and PfSUB3), MTRAP and PfDPAP2 are involved in PVM and erythrocyte membrane rupture, and aspartic proteases in gamete round-up (Munsamy et al., 2018). Egress is followed by exflagellation, mediated by the male-specific axoneme assembly (flagellum formation) proteins (e.g., the armadillo repeat protein PF16, Kinesin 8B, SAS-6 (Straschil et al., 2010; Depoix et al., 2020); MAP2 and CDC20 (Kumar et al., 2021); cytoskeletal proteins α-tubulin II and actin II (Elena et al., 2011) and *Pf*MR5 (Eksi et al., 2006). Mutual recognition and male and female gamete attachment are mediated by P230, Pfs48/45, Pfs47 (Van Dijk et al., 2010), complement control CCp proteins (Baker, 2010) and Pb115 (Liu et al., 2019) (**Figure 4**).

After fertilization, the resultant zygote develops into an ookinete with pronounced energy requirements through oxidative phosphorylation and the glycolytic pathway (Delves et al., 2019). HAP2 (Kuehn and Pradel, 2010) and protein disulphide isomerase (PDI) (Angrisano et al., 2019) are responsible for nuclear fusion, and genome tetraploidy in the resulting zygote is mediated by NIMA-related kinases, NEK-2 and NEK-4 (Pradel, 2007). The mature ookinete is motile and capable of cell invasion, which is mediated by glideosome machinery under the regulation of AP-O (Singh et al., 2021b) and involving cGMP mediated signaling (Gao et al., 2018) and kinases such as PKG, CDPK1, CDPK3, and PK7 (Singh et al., 2021a) (**Figure 4**).

### Targeting Parasite Development in the Mosquito

Since gametogenesis involves re-activation of processes that were targetable during ABS development (such as DNA replication) as well as male and female specific events, targeting of gametogenesis and oocyst formation have been the topic of much discussion. Indeed, several screening cascades places gamete formation or downstream oocyst formation as primary endpoints to identify transmission-blocking antimalarials, with the potential broader hit detection capacities compared to gametocytocidal screens that will not be able to detect compounds that do not affect their viability but only compromise mature gametocytes, thereby sterilizing them for downstream fertilization (Delves et al., 2019). In fact, the transmission-blocking activity of any compound active on mature gametocytes has to be validated on oocyst formation (Birkholtz et al., 2022). Importantly, this must consider natural oocyst densities, which then prioritizes development candidates M5717, MMV048 and KAE609 as the most effective for transmission-blocking (**Table 3**) (Dechering et al., 2017). Compounds with sole gametocidal or sporontocidal activity will be challenging to develop since they would need an extraordinarily long human serum half-life to still be effective once taken up into the mosquito vector. Strategies focused on direct delivery to mosquitoes is currently seen as only viable alternative (Paton et al., 2019).

**Table 3:** Summary of key antimalarials, front-runner and development compounds as well as investigative hits with *in vitro* activity (>70% inhibition, or < 5 µM IC_50_) against male and female gametes, as well as oocyst formation.

Compound	Gametes (%)^a^		TRA%^b^	Target	Ref
	Male	Female			
Chlorproguanil	–	–	100	DNA replication/ transcription	(Vos et al., 2015)
Cycloguanil	96	0			(Delves et al., 2013)
P218	4*	–	99		(Vos et al., 2015; Posayapisit et al., 2021)
Pyrimethamine	94	0	100		(Delves et al., 2013; Vos et al., 2015)
Atovaquone	12	12	100	Cytochrome bc1 (Qo)	(Delves et al., 2013; Vos et al., 2015)
ELQ-300			100	Cytochrome bc1 (Qi)	(Nilsen et al., 2013)
Cycloheximide	100^#^	–	100	Unknown	(Vos et al., 2015)
KDU691	–	–	100	*Pf*PI4K	(Mcnamara et al., 2013)
MMV390048	90*	–	69		(Paquet et al., 2017)
AZD-0156	84^&^		65^&^		(Reader et al., 2021)
M5717 (DDD498)	–	–	1.8*	*Pf*EF2	(Baragana et al., 2015)
GNF179	–	–	100	Protein secretory pathway	(Plouffe et al., 2016)
Ganaplacide (KAF156)	–	–	90		(Kuhen et al., 2014)
ML324	93^&^	–	95^&^	*Pf*Jmj3	(Reader et al., 2021)
JIB-04	10	80	–		(Matthews et al., 2020)
ML10	–	–	41*	*Pf*PKG	(Baker et al., 2017)
MMV030084	141*	0	–		(Vanaerschot et al., 2020)
Methylene blue	98	11	99	Glutathione reductase	(Delves et al., 2013; Vos et al., 2015)
Epoxomicin	–	–	100	β2 and 5 subunits of the proteasome	(Czesny et al., 2009)
BIX01294	120*	727*	–	HKMT	(Malmquist et al., 2015)
SJ733			5mg/kg^@^	*Pf*ATP4	(Dechering et al., 2017)
KAE609 (NITD609)			100		(Van Pelt-Koops et al., 2012)
Birinapant	75^&^		78^&^	Caspase 3	(Reader et al., 2021)
MMV1581558	79^&^		80^&^	Unknown (Adenosine A3 receptor)	
MMV1580843	70^&^		84^&^	Unknown	
SQ109	53^&^		80^&^	Unknown (MmpL3 in Mtb)	
TCMDC-137173	76	21	25*	Unknown	(Delves et al., 2019)
TCMDC-134114	99	0	56*		
DDD01035881	540*	>10*		*Pf*s16	(Delves et al., 2018b; Yahiya et al., 2021)
DDD01028074	220*	>10*		Unknown	(Delves et al., 2018b)

a % inhibition at 1 µM.

b TRA: transmission-reducing activity at 5 µM.

* IC_50_ (nM).

# % inhibition at 10 µM.

^&^ Inhibition of gamete formation at 2 µM.

^@^ ED_50_ in P. berghei.

As expected, signaling cascades associated with gametogenesis are inherently targetable, with PDE and PKG on the forefront, both being essential to gametogenesis and chemically targeted by Zaprinast, (Mcrobert et al., 2008) and imidazopyridines such as ML10 (Mcrobert et al., 2008; Taylor et al., 2010; Baker et al., 2017; Baker et al., 2020), respectively (**Table 3**). Ca^2+^ homeostasis is inherently targetable with several CDPKs having been investigated; CDPK1 with imidazopyridazines (Lemercier et al., 2009; Chapman et al., 2013; Large et al., 2013; Ansell et al., 2014; Chapman et al., 2014); CDPK4 by bumped kinase inhibitors (Huang et al., 2016) and pyrazolopyrimidine derivatives (Vidadala et al., 2014). This extends to CDPK3 and PK7 during fertilization, with other activities such as PDI and PDE also potentially important (Angrisano et al., 2019). Targeting energy metabolism is important in the metabolically active ookinetes and oocysts, as evident by sporonticides atovaquone and ELQ-300 as potent inhibitors of cytochrome bc1 (**Table 3**) and recent evidence also points to the importance of targeting cytoskeletal proteins such as *Pf*s16 (Yahiya et al., 2021) that plays a role during the immediate phase of microgametogenesis with DDD01035881 (Delves et al., 2018b).

## Conclusion and Future Directions

The vast expansion of our understanding of the processes mediating transmission and survival of malaria parasites over the past decade has provided a deep insight into the possibilities and challenges associated with the identification and development of transmission-blocking interventions. ‘Omics data clearly indicates the importance of transcriptional reprogramming that manifests as metabolic adaptations and is initiated during commitment but evidently established in immature gametocytes and progressing during maturation to result in differentiated sexual parasites. Although such ‘blueprints’ are inherently informative, we are far from understanding the regulatory mechanisms involved but we do understand the involvement of epigenetic, transcriptional, and post-transcriptional mechanisms. New developments in inducible gene manipulation systems will allow a deeper investigation of the importance of gametocyte-specific regulators (Tiburcio et al., 2019), and this information could be translated to identify druggable candidates for guided, target-based discovery of novel transmission-blocking chemotypes. Additionally, the use of proteomics to identify and validate drug targets in gametocytes have been developed (Dziekan et al., 2019; Yahiya et al., 2021), yet, we are far from a streamlined drug target identification and mode-of-action platform for gametocytes similar to the extensive pipeline used to characterize ABS actives (Yang et al., 2021). Indeed, if any of the new transmission-selective hits are to be further developed, this area needs investment and development. Knowledge of a transmission-blocking compound’s target is particularly needed to allow combinations with ABS actives with a different target to have any success in preventing spread of resistance. If we are successful in identifying unique transmission-blocking antimalarials, it is envisaged that they could be used as add-on to current therapeutic strategies in combination with ABS actives or can be used as stand-alone treatments to clear gametocyte carriers of parasites. Innovations such as alternative culture conditions for gametocytogenesis to more closely reflect the *in vivo* environment associated with *P. falciparum* gametocyte development; humanized mouse models of *P. falciparum* transmission to evaluate efficacy of transmission-blocking actives; adipo formulations for long-lasting release of gametocytocidal drugs, or compounds targeting the parasite when delivered directly to the mosquito vector, may indeed be promising to prevent the spread of malaria parasites and hence this disease.

## Author Contributions

L-MB conceived the work and MvdW, JR, and L-MB wrote and edited the paper. All authors contributed to the article and approved the submitted version.

## Conflict of Interest

The authors declare that the research was conducted in the absence of any commercial or financial relationships that could be construed as a potential conflict of interest.

## Publisher’s Note

All claims expressed in this article are solely those of the authors and do not necessarily represent those of their affiliated organizations, or those of the publisher, the editors and the reviewers. Any product that may be evaluated in this article, or claim that may be made by its manufacturer, is not guaranteed or endorsed by the publisher.

## Abbreviations

ABS, asexual blood stage; AP2-G, Apetala 2-G; CCp, complement control protein; CDPK1, calcium dependent protein kinase 1; EDC, exo-erythrocytic developmental cycle; EF2, Elongation factor 2; GDV-1, gametocyte development 1; HKMT, histone lysine methyltransferases; HP1, heterochromatin protein 1; IDC, intra-erythrocytic developmental cycle; IMC, inner membrane complex; GEXP, gametocyte exported proteins; MDV-1, male development protein 1; NPP, new permeation pathways; PEG3, protein of early gametocyte 3; PMT, phosphoethanolamine methyltransferase; STEVOR, subtelomeric variable open reading frame.
